# A multi-tiered approach for assessing the forestry and wood products industries’ impact on the carbon balance

**DOI:** 10.1186/s13021-015-0014-9

**Published:** 2015-02-03

**Authors:** Marcus Knauf

**Affiliations:** Knauf Consulting, Dorotheenstraße 7, D-33615 Bielefeld, Germany

**Keywords:** Forestry and wood products industry, Multi-tiered approach for assessing, Kyoto-protocol-oriented approach, Consumer-oriented approach, Carbon footprint, Value-creation-oriented approach, C-sink, Substitution, North Rhine-Westphalia

## Abstract

**Background:**

The forestry and wood products industries play a significant role in CO_2_ emissions reduction by increasing carbon stocks in living forest biomass and wood products. Moreover, wood can substitute for fossil fuels. Different methods can be used to assess the impact of regional forestry and wood products industries on regional CO_2_ emissions. This article considers three of those methods and combines them into a multi-tiered approach.

**Results:**

The multi-tiered approach proposed in this article combines: 1) a Kyoto-Protocol-oriented method focused on changes in CO_2_ emissions resulting from regional industrial production, 2) a consumer-oriented method focused on changes in CO_2_ emissions resulting from regional consumption, and 3) a value-creation-oriented method focused on changes in CO_2_ emissions resulting from forest management and wood usage strategies. North Rhine-Westphalia is both a typical German state and an example of a region where each of these three methods yields different results. It serves as a test case with which to illustrate the advantages of the proposed approach.

**Conclusions:**

This case study argues that the choice of assessment methods is essential when developing and evaluating a strategy for reducing CO_2_ emissions. Emissions can be reduced through various social and economic processes. Since none of the assessment methods considered above is suitable for all of these processes, only a multi-tiered approach may ensure that strategy development results in an optimal emissions reduction strategy.

## Background

### The forestry and wood products industries’ impact on climate protection and how it is reflected in climate reporting

Forests have a significant impact on the global carbon cycle and therefore on the climate [1]. On the one hand, they help improve local climates by moderating temperature and humidity, while on the other, they absorb atmospheric carbon (CO_2_) through photosynthesis and forest growth and store it for the long term (sequestration). When wood is used in furniture or construction, for example, the sequestered carbon remains stored in the wood product (wood carbon stock) [2-4]. Wood can substitute for fossil fuels such as oil, gas or coal. Not only can wood be burned for fuel (fuel substitution) [5-7], production and disposal of wood products typically require less energy than products made from other materials (material substitution) [8-15].

In the context of climate reporting, article 3.4 of the Kyoto Protocol recognizes an increase in the forest’s carbon stocks as a CO_2_ emissions reduction measure. Forest carbon sequestration is assessed within the framework of the “land use, land use change and forestry” (LULUCF) sector, cf. [16,17]. According to the IPCC Good Practice Guidance for Land Use, Land Use Change and Forestry [16,17] accounting must include the five carbon pools: (1) above-ground biomass, (2) below-ground biomass, (3) deadwood, (4) litter and (5) organic soil carbon [18]. These reports are included in national greenhouse gas inventories [19]. In the first commitment period of the Kyoto Protocol, from 2008 to 2012, the assumption was that “all carbon removed in wood and other biomass from forests is oxidized in the year of removal” [20]. However, this assumption did not take into consideration that wood removal does not result in an immediate release of CO_2_ [21,22]. The decisions made at the Conferences of the Parties in Copenhagen 2009, Durban 2011 and Doha 2012, necessitated a follow-up agreement to the Kyoto Protocol to address the sink function of harvested wood products (HWP) [23]. A forest management reference level (FMRL) [24,25] accounts for carbon stored in HWP. In climate reporting, the effects of substitution are recorded as CO_2_ reductions within the industry and energy sectors and are, therefore, not recognized as a contribution of the forestry and wood products industries.

To fully assess and increase the forestry and wood products industries’ contribution to climate protection, an integrated study of the forestry and wood products industries which accounts for all storage and substitution effects [1,14,26,27] is needed. Such a study could be conducted either at the national [14,28] or regional level [29].

### The Kyoto Protocol’s geographical approach and its limitations

Climate reporting under the Kyoto Protocol follows a geographical approach [30], assigning territorial responsibility for CO_2_ emissions to the producers (e.g. industry CO_2_ emissions). It allocates emissions to the emitter. This is also the case for the forestry and wood products industries. Studies which assess the overall impact of CO_2_ emissions on climate protection (see previous paragraph), follow the same geographical approach by observing a specific forest area (whether national or regional) and analyzing all of its associated carbon reduction effects (sequestration and substitution). Consumers are not included in this analysis. Many authors, however, have proposed including consumer effects in general, i.e. not specifically related to the forestry and wood products industries [31-36]. This argument is based on the premise that “the responsibility for carbon dioxide (CO_2_) emissions from economic activities lies with people’s attempts to satisfy certain functional needs and desires” [31]. This concept is generally referred to as the carbon footprint (CF) [37]. Current studies on the forestry and wood products industries’ impact on climate protection do not include the life cycle effects of consumption. Instead, the CF is only calculated at the individual products level [38]. However, following the premise that the forestry and wood products industries do in fact contribute to the reduction of CO_2_ emissions and thereby to climate protection, and also assuming that promoting wood use (e.g. increasing wood-based construction) is a sensible climate policy measure, then there is a deficit in the data needed to evaluate such measures in terms of consumer effects (e.g. CF). The CF is normally associated with CO_2_ emissions and thus accounted for as a debit. However, in the case of the forestry and wood products industries, the CF is typically associated with a reduction in CO_2_ and expressed as a credit. The following paper treats the CF of the forestry and wood products industries as a negative CF.

### The purpose of this paper is to present an analytical model combining three approaches

This paper presents a tool that accounts for the CO_2_-responsibility of consumers [32] in assessing the forestry and wood products industries’ impact on climate protection. It proposes an analytical model with three distinct approaches: 1) an approach based on the principles of the Kyoto Protocol and focused on emitters, 2) a consumer-oriented approach (carbon footprint) and 3) an approach based on the value chain of the forestry and wood products industries. A particular aspect of this analytical model with its three approaches is that the effects of wood usage (storage and substitution) are allocated differently. The paper presents its analytical model in the form of a generally accepted matrix that incorporates the four CO_2_ reducing effects of the forestry and wood products industries (forest sink, HWP sink, fuel substitution, material substitution). The model is then tested in a case study assessing the German state of North Rhine-Westphalia.

## Results and discussion

### Development of a universal model for assessing the forestry and wood products industries’ impact on the carbon balance

The three approaches developed for this study uniformly assess sequestration in the forest according to the IPCC Good Practice Guidance for Land Use, Land Use Change and Forestry [16-18] and in conformity with the international conventions of climate reporting. The approaches differ with respect to the assessment of wood usage and its related C-effects. The following three questions characterize the different approaches:

- ▪ Approach I (Kyoto-Protocol-oriented): “How do the forestry and wood products industries in Area x impact the CO_2_ balance in Area x (and the global CO_2_ balance)?”
- ▪ Approach II (consumer-oriented): “How do the forest and consumers in Area x impact the global CO_2_ balance?”
- ▪ Approach III (value creation-oriented): “How do the forest and harvested wood products from Area x impact the global CO_2_ balance?”

Approach I applies the Kyoto Protocol approach and concentrates on the emitter. In addition to sequestration in the forest, this model takes into account the HWP from Area x. The carbon stock levels in harvested wood products are calculated according to the IPCC classification [25]; this assessment model provides a country-specific method of calculation. It also takes CO_2_ emissions reduction through the substitution of fossil fuels into account. This model includes wood in Area x used as fuel (fuel substitution), as well as wood in Area x used to manufacture products (material substitution). The emissions reductions are calculated based on the volumes of wood utilized and manufactured into finished products by multiplying the mass C in wood (expressed as t C) with the underlying substitution factors. To assess fuel substitution, for example, the substitution factor is calculated from the difference in emissions between fossil fuels (e.g. defined mix) and wood, based on the carbon content of the wood utilized [14,39,40]. Excluding the fossil fuel consumption inherent in forest management, timber harvesting and transport, which makes up less than 10% of the total emissions profile, the use of wood for fuel is considered CO_2_ neutral, cf. [39,41]. Material substitution is assessed using the general substitution factors developed by [15]. Here the difference between CO_2_ emissions (expressed as C) of competing products (wood versus non-wood) with the same functionality are set in relation to their carbon content. In addition to general substitution factors, product or product-specific substitution factors can also be used, cf. [14,27]. The substitution effects are not visible per se but are reflected in the greenhouse gas inventory of Area x (if a regional greenhouse gas inventory has been conducted). They are expressed as CO_2_ emissions reductions in the energy and industrial sectors, but not attributed to the forest-based industries. The accounting method of Approach I makes it possible to identify the substitution effects as contributions of the forestry and wood products industries and evaluate them accordingly.

In contrast to Approach I, Approach II does not concentrate on CO_2_ emissions (e.g. of the industrial sector) or the CO_2_ emissions avoided through substitution, but instead focuses on the consumption of wood products in Area x. Approach II shows the CO_2_ effects associated with the wood products used in Area x (over their entire life cycle). The approach concentrates on the carbon footprint and offers a way to incorporate consumer responsibility into the analysis of the forestry and wood product industries’ impact on CO_2_ emissions reduction. It also prevents so-called leakage effects [42]. An assessment method that disregards the location of wood usage ignores any climate-conscious consumption and investment decisions impacting the CO_2_ footprint of a region or a country, rendering them irrelevant. The impact from fuel substitution can be measured the same way as in Approach I. The evaluation of the CO_2_ effects of wood products, however, differs in that Approach II links the wood products used to Area x. These wood products are taken into account when determining the carbon stock in wood products and assessing material substitution. The volume of utilized wood products is calculated using input-output analyses based on either official statistics [43], or empirical studies [44]. The material-substitution calculations are based on the utilized wood products and the substitution factor for the material substitution [15].

Approach III can be used as a basis for determining which forest management measures and wood usage strategies in Area x have the greatest impact on CO_2_ reduction. This approach is referred to as the value-creation approach because it emphasizes assessment of the effects of wood harvested in a region’s forests as part of the value chain in the forestry and wood product industries. Approach III provides useful information for designing the forest-wood value chain to optimize climate benefits. For example, this approach can be used to develop forest management scenarios for future forest development [29,45]. In Approach III carbon stocks in the forest and harvested wood are evaluated using the same methodology as in Approach I [18,25]. Calculating the fuel and material substitution levels requires a harvested wood products utilization model that takes into account current material flows (related to any fuel and material use). Fuel and material substitution levels are calculated based on this utilization model and substitution factors (see explanations under Approach I).

These three approaches are equally valid but can be used to draw different conclusions. While Approach III is favored for the development of climate-optimal strategies for the forestry and wood products industries, Approach II is primarily used when assessing of wood usage and Approach I is helpful in evaluating the local forest-based industry and its specific impacts. The paper sets the three approaches in relation to the four effects associated with the forestry and wood products industries’ contribution to climate protection (forest sink, HWP sink, fuel substitution, material substitution) to create a 12-field matrix and adds a totals column to form a 15-field matrix (Table 1).

**Table 1:** **Matrix for assessing the impact of the forestry and wood products industries on the CO**_**2**_ **balance (Area x)**

	**Category of emission reduction/stock/sink**				**TOTAL**
**Approaches for assessing the forestry and wood products industries’ impact on the CO**_**2**_ **balance (Area x)**	**Forest sink**	**HWP sink**	**Fuel substitution**	**Material substitution**	
	Changes in forest carbon stock	Changes in HWP carbon stock	CO_2_ impact of wood fuel	CO_2_ impact of wood product usage	
	**[Mt CO**_**2**_ **]**	**[Mt CO**_**2**_ **]**	**[Mt CO**_**2**_ **]**	**[Mt CO**_**2**_ **]**	**[Mt CO**_**2**_ **]**
***How do the forestry and wood product industries in Area x impact the CO***_***2***_ ***balance in Area x (and the global CO***_***2***_ ***balance)?***	Forest Area x	HWP from Forest Area x	All wood used as fuel in Area x	All wood processed/manufactured in Area x	Overall impact Approach I
**Approach I: Kyoto Protocol-oriented approach**					
***How do the forest and consumers in Area x impact the global CO***_***2***_ ***balance?***	Forest Area x	All HWP used in Area x	All wood used as fuel in Area x	All HWP used in Area x	Overall impact Approach II
**Approach II: Consumer-oriented approach**					
***How do the forest and the harvested wood products from Area x impact the global CO***_***2***_ ***balance?***	Forest Area x	HWP from Forest Area x	Wood from Area x used as fuel	HWP from Forest Area x	Overall impact Approach III
**Approach III: Value-creation approach**					

In a country without any international trade in timber or wood-based products, the differences in the three different approaches would be redundant, as all three approaches would yield the same results. A similar situation would arise if there were no foreign trade surplus in the trade of wood products. However, a country without forestry and wood product industries that only imports wood products would show a zero value in Approaches I and III (assuming it would not use any wood for fuel), while Approach II would yield a value based on the amount of imported wood (carbon stock in the wood and material substitution; the disposal of the wood products would have to be taken into account, however). A heavily forested country with strong forestry and wood products industries, a small population and high timber exports would exhibit a significantly higher value in Approaches I and III than in Approach II. Looking at all countries combined, the sum of all of the values under Approach I equals the sum of all of the values under Approach II.

### Applying the model to the German state of North Rhine-Westphalia

A model combining these three assessment approaches makes it possible to calculate the CO_2_ effects for any area based on data pertaining to its forestry and wood products industries and wood usage levels. In most cases such data is readily available at the national level or can be extrapolated to supplement existing data (see above; or below, under Methods). In addition to national commitments, a growing number of states (e.g. the German state of North Rhine-Westphalia, NRW) have incorporated climate protection targets into their own statutes [46]. NRW has created greenhouse gas inventories based on the principles of the IPCC [47]. So far, these greenhouse gas inventories do not include forestry in the LULUCF sector and have also excluded any substitution effects of the forestry and wood products industries. Consequently, they fail to provide information on the forestry and wood product industries’ impact on the CO_2_ balance and climate protection in North Rhine-Westphalia. Using North Rhine-Westphalia as an example, the following paper shows how effects at the state level can be evaluated by applying a model combining three approaches. The paper discusses methodological difficulties. North Rhine-Westphalia serves as a case study; the results are exemplary and methodically transferable to any region.

North Rhine-Westphalia is characterized by its urban centers (Ruhr area, Rhineland) and with nearly 18 million consumers is Germany’s most populous state. Considering the number of inhabitants, it has relatively little forested area (915,800 hectares) and low harvest levels (approximately 6 million m^3^ per year) [48]. However, the forest-based sector’s annual turnover of some EUR 35 billion (2010) [49] attests to the strong timber processing and wood manufacturing sector in NRW, which includes Eastern Westphalia, an important center for the German and European furniture industry and in particular kitchen manufacturing.

Table 2 shows the model results for North Rhine-Westphalia based on the matrix in Table 1. The values refer to the period from 2005 to 2010 and are expressed in units of Mt CO_2_ (conversion from Mt C using the conversion factor of 44/12 = 3.67). Table 2 shows CO_2_ reductions as negative values, as stipulated in international climate reporting guidelines. The Methods section specifies the exact calculation methods used. The values shown in Table 2 were determined on the basis of official statistics and empirical studies. The field “All HWP used in Area x” (Approach II), uses nation-wide data [44] because no regional statistics were available. The proportion of wood used was derived as a percentage of total wood use equal to the population of NWR expressed as a percentage of the total population. This assumption is justified because North Rhine-Westphalia is considered an average German state, with regard to relevant structural parameters for construction and wood usage (e.g., home ownership rates, income levels, building structures) [50]. For example, residents of NRW fall exactly in the middle of the range of purchasing power in Germany. Basing the analysis on national statistics presents a methodological weakness given the current lack of available data. This flaw makes it impossible to track individual developments in NRW in relation to the national average (for instance, to evaluate a program to increase wood use in construction or for heating with wood/pellets at the state level). However, it does not represent a basic or fundamental methodological weakness; it can be overcome if data from future empirical studies such as [44] become available not only for the national but also for the regional level.

**Table 2:** **Matrix for assessing forestry and wood products industries’ impact on the CO**_**2**_ **balance (North Rhine-Westphalia, Germany for the period from 2005 to 2010)**

	**Category of emission reduction/stock/sink**				**TOTAL**
**Approaches for assessing forestry and wood products industries’ impact on the CO**_**2**_ **balance (North Rhine-Westphalia, NRW)**	**Forest sink**	**HWP sink**	**Fuel substitution**	**Material substitution**	
	Changes in forest carbon stock	Changes in HWP carbon stock	CO_2_ impact of wood fuel	CO_2_ impact of wood product usage	
	**[Mt CO**_**2**_ **]**	**[Mt CO**_**2**_ **]**	**[Mt CO**_**2**_ **]**	**[Mt CO**_**2**_ **]**	**[Mt CO**_**2**_ **]**
***How do the forestry and wood product industries in NRW impact the CO***_***2***_ ***balance in NRW (and the global CO***_***2***_ ***balance)?***	Forest in NRW	HWP from forests in NRW	All wood used as fuel in NRW	All wood processed/manufactured in NRW	
**Approach I: Kyoto Protocol-oriented approach**	**(–4)**	**–1.1**	**–5.0**	**–7.9**	**–18.0**
***How do the forest and consumers in NRW impact the global CO***_***2***_ ***balance?***	Forest in NRW	All HWP used in NRW	All wood used as fuel in NRW	All HWP used in NRW	
**Approach II: Consumer-oriented approach**	**(–4)**	**–3.3**	**–5.0**	**–9.1**	**–21.4**
***How do the forest and the harvested wood products from NRW impact the global CO***_***2***_ ***balance?***	Forest in NRW	HWP from forests in NRW	Wood from NRW used as fuel (of which ca. 0.5 Mt is not statistically recorded as harvested wood)	HWP from forests in NRW	
**Approach III: Value-creation approach**	**(–4)**	**–1.1**	**–2.5**	**–3.6**	**–11.2**

Currently no regional data are available with which to calculate forest carbon storage levels. Therefore, a value was inferred based on the most recent national forest inventory in 2002 [51] (see Methods). This is a conservative estimate; the value is considered a placeholder until regional forest inventory data become available (BWI III or national forest inventory; 2015 or 2016). The value is given in parentheses to emphasize its statistical uncertainty.

North Rhine-Westphalia is a region with no congruency between forest areas, timber processing, wood product manufacturing and demand for wood products. That it is the most populous state in Germany is reflected in the value shown in Approach II of –21.4 Mt C, which is almost twice as high as the value determined for its own forest area and timber production (–11.2 Mt C, Approach III). The value in Approach I is relatively high, at –18 Mt C, when compared with the amount of timber sourced from its own forests. This value can be explained on the one hand by the fact that North Rhine-Westphalia has an extensive wood-products and furniture manufacturing industry and therefore allocates a value of –7.9 Mt C to material substitution. On the other hand, Approach I includes fuel substitution, to a large extent through wood burning in biomass incineration plants. Since this bioenergy use is partially coupled with the consumer markets through the recycled wood market or takes place within the wood products industry, Approach I yields a higher value than Approach III.

The values shown in Table 2 can be used as a benchmark for comparison with other areas (both states and countries) but the values need to be normalized. For Approach II it is suitable to refer to population levels and for Approach III to the forest area. For Approach I, it may make more sense, rather than using population as the reference, to draw a connection to a different economic indicator (e.g. economic output).

In principle, the values determined in Approaches I and II can also be set in relation to CO_2_ emissions in the corresponding area. The value of –18 Mt CO_2_ annually determined by Approach I can be compared, in accordance with the model, to the greenhouse gas emissions of NRW of 307 Mt CO_2_ (2010) [47]. The annual value of –18 Mt CO_2_ (about –1 t CO_2_ per capita) can therefore be interpreted such that the total CO_2_ emissions of North Rhine-Westphalia would be 18 million tons higher without the impact of the forestry and wood-products industries. This would represent a 5.9% increase in total greenhouse gas emissions for North Rhine-Westphalia in 2010. The value of –21.4 Mt CO_2_ in Approach II means that (global) CO_2_ emissions would be 21.4 million tons higher if the forest in North Rhine-Westphalia and the NRW consumers, by opting for wood products and wood energy, made no impact on the CO_2_ balance. The carbon footprint for NRW consumers would increase by 21.4 Mt CO_2_ (about 1.2 tons CO_2_ per capita) if the impact of the forestry and wood products industries were not considered. Assuming the current carbon footprint for NRW consumers of approximately 200 Mt CO_2_ (derived from the nationwide figures according to [42]), the result would be a 10.7% larger carbon footprint.

The value of 5.9% in Approach I is about half as high as the 10.7% in Approach II. Since in the past analysis of the forestry and wood products industries’ impact on the CO_2_ balance was assigned to national GHG emissions, it was only used as a reference in Approach I [52]. For a state such as North Rhine-Westphalia, however, such an approach is not appropriate. On the one hand, it does not adequately address the impact of wood usage; on the other hand, it is necessary to adjust the reference framework. While Approach I systematically uses the total GHG emissions as a reference, Approach II uses the carbon footprint. Approach II provides a consistent basis with which to integrate the overall carbon footprint of NRW consumers. This observation is particularly relevant for a state such as North Rhine-Westphalia. Nearly 22% of the population of Germany lives in North Rhine-Westphalia, and yet the state accounts for approximately 30% of German greenhouse gas emissions. The heavy industrial sector in North Rhine-Westphalia and the production of energy (mainly from fossil fuels) which provide products and energy for both the German and international markets create higher than average emissions levels for NRW [47]. Against this background, it is important to keep in mind that a resident of North Rhine-Westphalia with statistical CO_2_ emissions of 15 to 16 t CO_2_ per year is not 1.5 times more detrimental to the climate than the average German citizen (with CO_2_ emissions of approximately 10 t CO_2_ per year). The inclusion of these very important parameters is only possible if, as is done in Approach II, the carbon footprint is systematically evaluated.

## Conclusions

The multi-tiered approach presented in this paper with three distinct assessment approaches enables a more comprehensive analysis of the forestry and wood products industries’ impact on the CO_2_ balance. The model can be modified to meet specific objectives (e.g. to evaluate climate policy measures or to develop a basis for optimizing the forestry and wood products industries’ contribution to climate protection). Models like the one presented here, which allow for differentiation, pose a risk of being manipulated as a means to support a specific position. A region could, for instance, choose the model that yields the highest values in order to present its climate impact in the best possible light. The highly populated NRW region may select Approach II while the heavily forested region B may opt for Approach III, etc. This practice is an abuse of the model. However, given the considerations presented in this article, this abuse can be discovered and evaluated accordingly. Therefore, instead of promoting random and embellished conclusions (as is often the case when certain assessments are cherry picked to fit a respective view), the methodology in this study aims to explicate its results. The approaches presented in this paper aim to avoid obfuscated results by ensuring that the analysis of the forestry and wood product industries’ impact on climate change is carried out in a logical and consistent manner as proposed in Table 1.

The proposed methodology may at times be limited due to a lack of statistical data. At the national level these data are usually available (depending on the quality of official statistics) or can be derived from existing empirical studies. Regional statistics, however, are often lacking, and as a result the flow of goods between different regions (e.g. between different German states) within a larger territory (e.g. Germany) are poorly documented. This means that studies are left to rely on empirical data or national statistics, as is the case for all three assessment approaches described in this paper.

The underlying assumptions presented in this publication can be improved through in-depth analysis, such as: a) studies on wood use over the wood’s entire life span, including disposal; b) more in-depth studies on wood fuel (i.e., thermal heat, thermal heat extraction in power plants, domestic woodstoves, industrial and commercial wood fuel usage, efficiency); c) achieving greater accuracy in determining a region’s fuel substitution by defining a regional-specific fossil energy mix and a specific energy recovery key; d) achieving greater accuracy in assessing material substitution through more in-depth research (with regard to wood use, but also in terms of the comparison between products or product groups with the same functionality). The extent and depth of such additional studies should depend on the desired level of accuracy weighed against the significantly higher amount of time and effort they require.

Further studies are needed on how to allocate avoided CO_2_ emissions in the process chain in cases where semi-finished wood products are initially processed in region A and their end product is finished in region B. Approach I assesses material substitution proportionally in relation to the process chain. At the same time, the simplifying assumption is made that the substitution factors used are universally based on [15]; in this case, more factors specific to product groups could be used in the future [14,27].

## Methods

In addition to the explanation presented above under “Results and discussion,” the following illustrates in a structured manner how an analysis can be conducted at the national level (or for an area with well-defined statistics, e.g. the EU). Germany is used as an example and the assessment data is derived from official statistics and empirical studies available in Germany. The explanation is intended only as an overview. Finally, a detailed description is given of how the model was applied to the case study (NRW) presented in this paper. Possible problems arising from the processing of the data are discussed.

### Basic analysis

The matrix in Table 1 has eight different fields, which can be defined as areas of study; they are shown in Table 3.

**Table 3:** **Differentiation of the areas of study in the matrix for assessing the forestry and wood-products industries’ impact on the CO**_**2**_ **balance**

	**Category of emission reduction/stock/sink**			
**Approaches for assessing the forestry and wood products industries’ impact on the CO**_**2**_ **balance (Area x)**	**Forest sink**	**HWP sink**	**Fuel substitution**	**Material substitution**
	Changes in forest carbon stock	Changes in HWP carbon stock	CO_2_ impact of wood fuel	CO_2_ impact of wood product usage
	**[Mt CO**_**2**_ **]**	**[Mt CO**_**2**_ **]**	**[Mt CO**_**2**_ **]**	**[Mt CO**_**2**_ **]**
***How do the forestry and wood product industries in Area x impact the CO***_***2***_ ***balance in Area x (and the global CO***_***2***_ ***balance)?***	Forest Area x	HWP from Forest Area x	All wood used as fuel in Area x	All wood processed/manufactured in Area x
**Approach I: Kyoto Protocol-oriented approach**	**I**	**II**	**IV**	**VI**
***How do the forest and consumers in Area x impact the global CO***_***2***_ ***balance?***	Forest Area x	All HWP used in Area x	All wood used as fuel in Area x	All HWP used in Area x
**Approach II: Consumer-oriented approach**	**I**	**III**	**IV**	**VII**
***How do the forest and the harvested wood products from Area x impact the global CO***_***2***_ ***balance?***	Forest Area x	HWP from Forest Area x	Wood from Area x used as fuel	HWP from Forest Area x
**Approach III: Value-creation approach**	**I**	**II**	**V**	**VIII**

To begin with, a substitution factor is determined for material substitution SF_Ma_ and fuel substitution SF_Fuel_. SF_Fuel_ is used as a multiplier in calculating fields IV-V and SF_Ma_ is used as a multiplier in calculating the fields VI-VIII, for example a universal substitution factor SF_Ma_ can be taken from [14,27] and SF_Fuel_ can be taken from [14,27]. For the purpose of the study, universal means that the total wood volume was not assigned to different products or product groups, but rather that the same substitution factor can be used for all of the wood included in the study. For Germany, the universal substitution factors used are SF_Ma_ = 1.50 tC/tC and SF_Fuel_ = 0.67 tC/tC because the timber market and the distribution of products and product groups has already been taken into account in their calculation [27]. The substitution factor multiples for the C content of wood products or products used for fuel show the carbon mitigation of the wood use. In this sense the substitution factor can be described as a multiplier for calculation on the basis of C content in wood products.

Overview of the calculations for the eight areas of study:

I. Forest Area x:
  Based on national forest inventory studies [51], or on the basis of the LULUCF values reported in national greenhouse inventories [19].
II. HWP from Forest Area x:
  Based on a country-specific model according to [25] (see also [53]). Alternatively, an empirical input-output model can be applied, as for example by [44] (empirical determination of the inputs and outputs via analysis of disposal statistics).
III. All HWP used in Area x:
  Based on official statistics (net balance between data on production statistics and international trade statistics) or aggregated in wood balances, e.g. [54], or from empirical studies, e.g. [44].
IV. All wood used as fuel in Area x:
  Volumes are calculated based on empirical studies [55,56] or aggregated [57], or supplementary data e.g. from [58]; SF_Fuel_ = 0.67 tC/tC is used as multiplier.
V. Wood from Forest Area x used as fuel:
  Volumes are calculated based on a material-flow model, tracking the flow from timber harvesting (e.g. based on official harvest statistics) to end product [59]; supplementary data on harvested wood not included in official statistics, usually harvested for fuel (e.g. [60]) or as a balance of raw timber balances (e.g. [61]) and wood balances, (e.g. [54]); SF_Fuel_ = 0.67 tC/tC is used as multiplier; alternatively, using additional evaluations of the energy balance [62].
VI. All wood processed and manufactured in Area x:
  To determine wood volumes, see II.; SF_Ma_ = 1.50 tC/tC used as multiplier.
VII. All HWP used in Area x:
  To determine wood volumes, see III.; SF_Ma_ = 1.50 tC/tC used as multiplier.
VIII. All HWP from Forest Area x:
  To determine amounts, see V.; SF_Ma_ = 1.50 tC/tC used as multiplier.

### Analysis: NRW

The same eight fields are used as those in Table 3. They are also defined as areas of study and calculated as follows:

I. Forest Area NRW:
  No current statistical data exists for calculating the carbon stock levels in the forests of North Rhine-Westphalia. The forest carbon stock is determined based on forest inventories carried out every 10 to 15 years in Germany [51]. The last national forest inventory was conducted in 2002. The data collected retrospectively for the regional analysis is older, also at the time of the inventory due to the methodology used (for the period 1987-2002). A 2008 interim inventory [63] with reduced sample size provides only national and no regional data. Data are expected to be available for North Rhine-Westphalia in 2015 following the 3^rd^ National Forest Inventory (BWI III). A provisional (and thus given in parentheses) value for the year 2010 was determined on the basis of a simulation for the period of 2002-2010 [64]. The modeling of stock development since 2002 is subject to significant uncertainty, due in part to storm damage in 2007 (storm Kyrill). The recorded value is consistent with the calculations made by [65]. With the publication of the 3^rd^ National Forest Inventory in 2015 and a statewide forest inventory, probably in 2016, data will become available that can be used to accurately calculate stock levels for the period 2002-2012. There is discussion about conducting less expensive, modified inventories covering shorter periods to ensure timely and consistent monitoring of the forest stock (for example, with a tool used in North Rhine-Westphalia called “virtual forest” [66]).
II. HWP from forests in North Rhine-Westphalia (sink):
  A wood usage key was created using official statistics on the wood harvested from North Rhine-Westphalia. In addition, a material flow model was developed (based on impact statistics) that tracks wood processing from harvest to the end product. This study was carried out in accordance with [59] by distinguishing between softwood and hardwood varieties (because they have different timber yield levels and uses as industrial wood). The state forestry service made a distinction between logs and industrial wood based on consumer demand, which deviated from the national figures. An average value for the years 2005 to 2010 was calculated. The value provides a more accurate record of annual forest stock levels by taking in to account values distorted by storm damage in 2007 (storm Kyrill). Carbon storage levels in HWP were calculated based on the IPCC approach [25]. This approach determines the input of wood products into the HWP carbon storage pool and calculates the withdrawal from the product store (products with long, medium and short life-span (e.g. paper) as well as fuel wood) on the basis of a mathematical function (exponential decomposition curve).
III. All HWP used in NRW (sink):
  In contrast to II, the carbon storage in wood products used in NRW is calculated using an input-output measurement. The measurement was carried out using a modified model based on the nationwide material-flow model from [44] for the year 2007; the share attributed to NRW was derived based on the state’s share of the overall German population (21.8%); cf. “Results and discussion”.
IV. All wood used as fuel in NRW:
  Fuel substitution was quantified based on official statistics and various studies on bio-energy and renewable energy. The reporting recognizes the use of solid biomass (wood) for generating electricity. Since its intent is to promote bioenergy through renewable energy regulations (EEG) [67], the reporting has a relatively high level of transparency [39,58]. The much more extensive use of bioenergy for generating heat is significantly less transparent [68]. For the electricity sector, data are available at the state level [58], and thus for NRW (1.3 billion kWh). For the heating sector, these data were derived from nationwide data and supplementary studies. On the basis of [68], the calculations were structured into the following three areas: a) biogenic solid fuels used in households (share of NRW 8.5%; based on [56]: 6.8 billion kWh); b) biogenic solid fuels used in industry (share of NRW 19.5%; based on a comparison between [69] and [70]): 4.6 billion kWh); c) biogenic solid fuels used in heating plants and from heat extraction (percentage NRW 15.2% [58]: 1.0 billion kWh). The total value of 12.3 billion kWh is derived from the 2010 calculations of biomass heat generation in NRW. Based on the conversion numbers of the energy content in the CO_2_ emissions (GHG emissions) set by [68], a CO_2_ reduction (energy substitution) of 5.0 Mt CO_2_ was calculated for 2010.
V. Wood from forests in NRW used as fuel:
  Emissions reduction levels resulting from the use of NRW wood for fuel are quantified for the period of 2005-2010 (according to II.). The analysis is based on the following assumptions: a) 100% of the fuel wood reported in the official harvest statistics is used as fuel; b) 100% of the by-products from wood harvesting and processing that are not used as material for product manufacturing are used as fuel wood; c) 50% of the bark is used as fuel; d) 85% of industrial wood used in the paper industry is used as fuel energy at the end of the paper’s life cycle (according to experts from the pulp and paper industry). Given the steady paper consumption since 2000, it was assumed that the use of fuel wood is consistent over time; e) 68% of the recycled wood taken from the HWP sink is used as fuel (according to experts, taking into account the losses in the utilization phase and the recycling ratio, cf. [61]); f) based on expert estimates 700,000 to 800,000 m^3^ of wood is removed each year from NRW forests and not included in the official harvest statistics; this wood is presumably used as fuel. Emissions reduction was calculated using the substitution factor SF_Fuel_=0.67 tC/tC.
VI. All wood processed and manufactured in NRW:
  The calculation was carried out in three steps:
  1. Comparison of German and North Rhine-Westphalia energy balances [69,70]. Comparing energy usage for two relevant product groups from the forest-based industry – wood product (WZ 16) and furniture (WZ 31) manufacturing. The proportion of energy usage by the wood products and furniture manufacturing industries in NRW compared to the industry total in Germany is determined to be 22.1%. The calculated proportion roughly corresponds to the North Rhine-Westphalian population’s share of the German population (21.8%).
  2. The proportion of wood processed and manufactured in NRW is quantified based on finished product statistics, such as those found in [44] (proportionate to the population; cf.) III).
  3. Emissions reduction is calculated using the substitution factor SF_Ma_ =1.50 tC/tC. Calculating material substitution in this study area poses a methodological problem. Currently, a scientifically accurate assessment of material substitution is only possible at the end-product level. The manufacturing process leading to these end products includes many stages, and only the sum of these stages can be used to determine material substitution values. Therefore, assessing only the end products manufactured in North Rhine-Westphalia (e.g. according to official manufacturing statistics) does not yield an accurate figure, since many of the manufacturing stages leading to the finished products take place outside of North Rhine-Westphalia (for both wood and non-wood products). At the same time, steps in the manufacturing process performed within the North Rhine-Westphalian wood products industry contribute to the production of end products outside North Rhine-Westphalia. Therefore, the energy balance calculation relies on official statistics. The application of this procedure must meet the following conditions: (1) the energy consumption statistics are properly recorded. (2) the share of energy consumed in specific sectors (e.g. woodworking or carpentry) is the same as in the industrial sectors. (3) the plants and equipment used by companies in North Rhine-Westphalia and Germany are equal in terms of energy consumption. (4) the production methods in the wood product and furniture manufacturing industries are comparable; that is, they include similar production steps and products. These four conditions only partially apply. Since the substitution factors for specific wood product groups in the construction industry [27] are roughly the same as those for product groups in the furniture manufacturing sector, using this methodological approach to generate estimates is justified. This approach is currently best suited to making statements about the material substitution performance of a region’s wood products industry.
VII. All HWP used in NRW:
  For quantification of wood volumes, see III; emissions reduction was calculated using the substitution factor SF_Ma_ =1.50 tC/tC.
VIII. HWP from NRW forests:
  For determining the amounts, see V.; emissions reduction was calculated using the substitution factor SF_Ma_ =1.50 tC/tC.
